# Socioeconomic Inequalities in Frailty in Hong Kong, China: A 14-Year Longitudinal Cohort Study

**DOI:** 10.3390/ijerph17041301

**Published:** 2020-02-18

**Authors:** Ruby Yu, Cecilia Tong, Jason Leung, Jean Woo

**Affiliations:** 1 Department of Medicine and Therapeutics/Jockey Club Institute of Ageing, The Chinese University of Hong Kong, Shatin, Hong Kong; jeanwoowong@cuhk.edu.hk; 2 Jockey Club Institute of Ageing, The Chinese University of Hong Kong, Shatin, Hong Kong; ceciliatong@cuhk.edu.hk; 3 Jockey Club Centre for Osteoporosis Care and Control, The Chinese University of Hong Kong, Shatin, Hong Kong; jason-leung@cuhk.edu.hk

**Keywords:** subjective social status, socioeconomic status, inequalities, frailty, ageing

## Abstract

The prevalence of frailty varies among socioeconomic groups. However, longitudinal data for the association between subjective social status and frailty is limited. In this study, we examined whether subjective social status was associated with incident frailty. Data were obtained from a 14-year cohort of Chinese men and women (n = 694) aged 65 years and older who participated in the MrOs study—a longitudinal study on osteoporosis and general health in Hong Kong. Subjective social status at baseline (2001–2003) was assessed using a 10-rung self-anchoring scale. Incident frailty at the 14-year follow-up (2015–2017) was defined as proposed by Fried and colleagues. Ordinal logistic regressions were used to examine the association between subjective social status (high, middle, or low) and incident frailty. After adjustment for age, sex, marital status, objective socioeconomic status, medical history, lifestyle, mental health, and cognitive function, subjective social status at baseline was negatively associated with risk of developing frailty over time (OR 2.3, 95% CI 1.2–4.6). In sex-stratified analysis, the social gradient in frailty was only found in men. Social inequality in frailty in men but not in women supports interventions specific to gender inequality and frailty.

## 1. Introduction

Frailty is a state of increased vulnerability. People who are frail are at greater risk of many adverse health outcomes, including falls, disability, and mortality [1,2]. On this basis, the World Health Organization (WHO) identified frailty as an international priority area for concerted action [3]. In England, identification of frailty was made a contractual requirement for all primary care practices since 2017 [4,5,6]. Many predisposing factors of frailty have been identified, including older age, being female, unhealthy behavior, underweight, low muscle strength, polypharmacy, cognitive impairment, and mood disorders [7,8,9]. Interventions targeting frailty, either by resistant exercise or nutrition supplementation have also been demonstrated to be effective in preventing or reducing frailty in many ageing societies around the world [10]. 

While research into frailty is burgeoning, the importance of socioeconomic factors as predictors of frailty and impact has seldom be considered, although low socioeconomic status (SES) is one of the strongest predictors of morbidity and premature mortality worldwide [11,12]. Indeed, the prevalence of frailty varies among socioeconomic groups – Individuals with lower SES, objectively measured by education, income, or occupation, have higher levels of frailty than those with high SES [7,13,14,15,16]. A limited number of longitudinal studies have also demonstrated that low SES increases the risk of frailty [17,18,19,20,21], but subjective measures of SES have rarely been used in association with frailty. There is evidence that subjective SES (e.g., subjective social status), which reflects the cognitive averaging of standard markers of SES [22] and captures psychological factors related to objective socioeconomic measures [23], may be more powerful in examining social determinants of health than objective measures alone [24,25]. Furthermore, there is evidence of the adverse effects of low subjective social status for physical performance, health-related quality of life [26], functional decline [27,28], and mortality [29] in older people. However, subjective social status has not been used to predict incident frailty.

To inform public health policy to prevent or delay the progression of frailty, there is still a need for longitudinal evidence to delineate association between lower SES and frailty. Using data from a 14-year longitudinal Chinese cohort, we examined whether subjective social status was associated with incident frailty.

## 2. Materials and Methods

### 2.1. Participants

The MrOs (Hong Kong) study, a cohort study on osteoporosis and general health in Hong Kong, began in 2001–2003 (Baseline). It involves a community sample of men and women aged 65 years and over. Recruitment notices were placed in elderly community centres and housing estates and talks given explaining the proposal of the study. Quota sampling using non-random recruitment was used to recruit approximately the same number of people in each of the three age strata: 65–74, 75–84, 85+ years. Those who were unable to walk independently, had bilateral hip replacement or were not competent to give informed consent were excluded. MrOs collects information on demographics, SES, health, lifestyle, and well-being of older people. Further details about MrOs are published elsewhere [30]. Participants were interviewed in the study site at the Prince of Wales Hospital at baseline, year 2, year 4, and year 14 by trained interviewers. The study was approved by the Clinical Research Ethics Committee of the Chinese University of Hong Kong, which required informed consent to be obtained. All participants signed the study consent form. The study is reported according to the STROBE statement.

### 2.2. Exposure

Subjective social status was assessed using a 10-rung self-anchoring scale. Participants were asked to place a mark on a picture of an upright ladder with 10 rungs, with the lowest rung indicating the most undesirable and the highest rung indicating the most desirable state with respect to their standing in the community (community status ladder). This is a subjective measure of social status developed by the John D. and Catherine T. MacArthur Research Network on Socioeconomic Status and Health, and has been associated with key health outcomes in various population surveys of different cultural and ethnic groups [31]. The ladder represents the individual’s perception of his/her status in the community.

### 2.3. Outcome

The outcome of the study was incident frailty which was assessed using the 5-item Cardiovascular Health Study (CHS) frailty phenotype, with total scores ranging from 0 to 5 [1]. The five items are unintentional weight loss, self-rated exhaustion, weakness (grip strength), slow walking speed, and low physical activity. The equivalent variables used in this study for the construction of the CHS score were body mass index (BMI) less than 18.5 kg/m^2^, having no energy, grip strength measurement in the lowest quartile, walking speed measurement in the lowest quartile, and Physical Activity Scale of the Elderly (PASE) score in the lowest quartile. The total scores were used to categorize participants as robust (score = 0), pre-frail (score = 1–2), and frail (score = 3–5). Participants who were classified as pre-frail or frail at baseline were excluded from the analysis. 

### 2.4. Covariate

Information on demographics, objective measures of SES, medical history, lifestyle, health-related quality of life (mental component), and cognitive function was collected using a questionnaire at baseline and follow-up, administered by an interviewer in private. BMI was also obtained.

#### 2.4.1. Demographics

Demographics (age, sex, marital status) were obtained by self-report.

#### 2.4.2. Objective Measures of SES

Objective measures of SES (educational level, maximum life-time income) were obtained by self-report.

#### 2.4.3. Medical History

Physician-diagnosed disease (hypertension, diabetes, stroke) was obtained by self-report.

#### 2.4.4. Lifestyle

Smoking status and alcohol consumption were obtained by self-report.

Physical activity level was assessed using PASE [32]. This is a 12-item scale measuring the average number of hours per day spent in leisure, household, and occupational physical activities over the previous 7-day period. Activity weights for each item were determined based on the amount of energy expended, and each item score was calculated by multiplying the activity weight by activity daily frequency. A summary score of all the items reflected the daily physical activity level.

The Diet quality index-International (DQI-I) was used to assess the quality of diet [33], which has been used to evaluate the quality of diet in a Chinese population [34]. Four major aspects of the diet are assessed: variety, adequacy, moderation, and overall balance, each with subcomponents. The range is 0–100 with higher scores indicating higher quality.

#### 2.4.5. Health-Related Quality of Life (Mental Component)

Health-related quality of life was assessed using the Short Form-12 (SF-12) [35], which has been validated in a Chinese population aged 65 years [36]. The Mental Component Summary (MCS) score was used in the analysis.

#### 2.4.6. Cognitive Function

Cognitive function was assessed using the Mini-Mental Status Examination (MMSE) [37], which has been validated in a Chinese population [38]. It is a 30-item questionnaire composed of multiple domains of cognitive function, including tests of orientation to time and place, registration, attention and calculation, recall and language and praxis. MMSE score ranges from 0 to 30 with lower scores reflecting more dementia-related cognitive impairment.

### 2.5. Data Analysis

Baseline characteristics of participants of both sexes were compared across subjective social status groups at baseline and frailty groups at year 14 using analysis of variance (ANOVA) or Chi-squared tests. The scores for the community status ladder were grouped into high (≥7), middle (5–6), or low (<5) according to the distribution of the scores. PASE, DQI, BMI, SF-12 MCS, and MMSE were treated as continuous variables. Ordinal logistic regression models were developed to examine the relationships of incident frailty with subjective social status (community status ladder), adjusting for age and sex (model 1), marital status (model 2) and then objective measures of SES (educational level and maximum life-time income) (model 3) to examine the relative contributions of objective measures to the relationship between subjective social status and frailty. Additional regression models were developed with further adjustment for smoking status (non-current smoker or current smoker), alcohol consumption (non-current drinker or current drinker), PASE, DQI, BMI, SF-12 MCS and MMSE (models 4–7). Covariates used for analysis were baseline data. The analysis was then repeated using educational level, and maximum lifetime income as the exposure to predict incident frailty, and separately for women and men. All statistical analyses were performed using the statistical package SPSS, version 24 (SPSS, Inc., Chicago, IL, USA). *P* values less than 0.05 were considered statistically significant.

## 3. Results

The original sample at baseline comprised 4000 participants, 1659 and 203 of whom were classified as pre-frail and frail and were excluded from this study. Of the 2138 robust participants, 694 (32.5%) had baseline data for subjective social status and follow-up data for frailty at year 14, and these were included in the analytical sample. Of the 694 participants who were retained in the final analysis, 419 and 211 developed pre-frailty and frailty at year 14, respectively.

Table 1 presents the baseline characteristics of the analytical sample according to subjective social status (community status ladder) at baseline and frailty status at year 14. Participants from the low subjective social status were more likely to be a male and a current drinker compared with those from higher status. The incidence of frailty was greater in participants who were older, female, not married, and who had lower education, and lower maximum lifetime income. Frail participants, compared with their less frail counterparts, were also more likely to have hypertension and diabetes, a higher BMI, and a lower MMSE score at baseline.

Table 2 shows the distribution of the study population on the subjective and objective measures of SES. There was no significant association between subjective social status (community status ladder), educational level, and maximum lifetime income for both men and women.

Table 3 shows the adjusted ORs of subjective social status (community status ladder) on incident frailty. In model 1, low subjective social status compared to high subjective social status at baseline was associated with a higher risk of developing frailty (OR 2.4, 95% CI 1.3–4.4). After adjustment for marital status (model 2), educational level and maximum life-time income (model 3), medical history (model 4), lifestyle (model 5), mental health (model 6), and cognitive function (model 7), the associations remained significant, with ORs for those with middle subjective social status being 2.0 (95% CI 1.4–3.0) and those with low subjective social status being 2.3 (95% CI 1.2–4.6). However, objective measures of SES (i.e., educational level, maximum lifetime income) (Supplementary Tables S1a–2c) were not related to incident frailty.

Table 4 and Table 5 present the adjusted ORs of subjective social status (community status ladder) on incident frailty by sex. The results showed that subjective social status was a significant predictor in all models for men. For men, low (OR 3.6, 95% CI 1.5–8.4) and middle (OR 2.2, 95% CI 1.3–3.8) subjective social status compared to high subjective social status at baseline were associated with a higher risk of developing frailty. For women, subjective social status was not associated with incident frailty.

## 4. Discussion

This study demonstrates that in a 14-year follow-up cohort of older Chinese people, there was a social gradient in the development of frailty, where lower subjective social status at baseline was associated with a higher incidence of frailty after an average of 14 years for men only. These inequalities persisted after adjustment for demographics, educational level, maximum life-time income, medical history, lifestyle, mental health, and cognitive function.

Our results show a clear gradient in incident frailty for older people that runs from the highest to the lowest subjective social status. These results are consistent with those of other studies, which report that there is no evidence of threshold regarding social position and health [39]. A recent publication on Dutch data classified older people into groups according to their educational level and household income and found that older people with a low educational level or a low income level had higher odds of being frail during 13 years of follow-up compared with their counterparts [18,21]. A similar relation between socioeconomic position and frailty trajectories has been shown in 10 European countries [20]. However, our study is the first to document the socioeconomic inequalities in incident frailty using a subjective measure of social status. There is evidence that subjective social status may be more powerful in examining social determinants of health than objective measures alone [24,25]. Our previous study demonstrated a social gradient in subjective social status for physical performance and health-related quality of life, independent of objective socioeconomic measures [26]. Hence, findings of this study provide further support to the importance of the psychosocial processes relative social comparison [40]. 

In addition to objective measures of SES, a range of conventionally predisposing factors of frailty, including hypertension, diabetes, stroke, unhealthy lifestyle, poor mental health, and poor cognitive function was considered to understand how subjective social status affects the development of frailty. It was expected that adjustment for these factors would attenuate the association between subjective social status and incident frailty. However, subjective social status maintained an independent association with incident frailty. Therefore, it seems very likely that the subjective social status is an independent predictor of incident frailty, possibly as a measure of facets of social position not captured by the objective SES. The findings also suggest that social comparisons among older people are not based on the objective SES whereas other factors such as lifetime achievement, capabilities, and autonomy, as well as availability and quality of familial relationships and social networks may be more important in later life. Studies are required to further examine this aspect.

Subjective social status predicts incident frailty for men but not for women. This gender difference may represent different cultural responses to SES due to the different social norms or society’s expectations for men and women. Early studies have also documented gender stereotyping attitudes - for example, men are supposed to be agentic and avoid weakness, and women are supposed to be communal and avoid dominance [41,42]. Therefore, it may be that men are more likely to place a greater emphasis on their role as a financial provider and have a higher perceived importance of being ‘on top’, both of which could increase the likelihood of becoming distressed and having dissatisfaction in life with low subjective social status which in turn increases vulnerability to frailty.

Alternatively, the gender difference may be explained by the fact that women tend to rate themselves higher than men using the community status ladder, and that they have healthier lifestyle compared with men [26], therefore attenuate the effects of subjective social status on frailty in women. Our finding is consistent with those of another longitudinal study in the Netherlands, which report that socioeconomic inequalities in frailty were mainly present in men but not in women [21]. Another explanation might be that medical factors may be more relevant than subjective social status as an explanatory factor for incident frailty among older women. In this study, the presence of hypertension at baseline predicted incident frailty in women, independent of subjective social status, age, and other covariates. A number of other studies have also suggested that gender difference in socioeconomic health inequalities varies according to life stage and health measure [43]. However, the small number of women who ranked themselves low on the ladder could also have attenuated the observed association.

To attenuate the social gradient in frailty of older people, especially among men, as well as to reduce the number of frail older people, strategies to raise the perception of social status are needed. There is evidence that older people’s involvement in decision-making leads to tangible improvements in their self-esteem and to a stronger sense of empowered achievement [44,45] which may lead to a higher subjective social status. Furthermore, having multiple social roles such as grand-parenthood, employment, and volunteering may increase subjective social status among older people [46]. However, there are cultural differences in role experiences. Interventions to raise older people’s subjective social status should take into account cultural factors. Another possible way to increase subjective social status for older people is tackling societal ageism. Nevertheless, the public health importance of subjective social status in women should not be underestimated as women tend to experience higher levels of frailty than men [47].

This study has several strengths and limitations. One of the strengths is that the longitudinal design allows us to estimate the risk for incident frailty over 14 years. Besides, a broad range of factors covering medical history, lifestyle, mental health, and cognition were collected allowing us to examine multiple potential mediating pathways that link subjective social status and incident frailty. However, the effect of subjective social status on incident frailty might have been underestimated as the attrition of the sample was significant, given that incident frailty and death are likely to be highly related. Baseline comparisons of those lost to follow-up and those retained in the follow-up found that those lost to follow-up were significantly older, more often not married, less educated, with lower lifetime earnings, lower subjective social status, and were frailer (Supplementary Table S3). Furthermore, information on current income was lacking and maximum life-time income was subject to recall bias. Early-life SES was also unknown although associations between early-life SES and a broad range of health outcomes have been documented extensively [48]. Additionally, all covariates except cognitive function (as measured by MMSE) and BMI were self-reported which are susceptible to systematic reporting bias. Finally, the use of MMSE for delineation of cognitive function may under-diagnose some individuals with cognitive impairment and early dementia, in particular highly educated individuals, since MMSE scores correlated with years of education.

## 5. Conclusions

In conclusion, this study provides evidence for a social gradient in the development of frailty where low subjective social status could lead to increased risk of frailty independent of objective measures of SES and other predisposing factors of frailty for men. This finding points out the importance of integrating social determinants of health, particularly the subjective dimension of SES, into the prevention and monitoring of frailty trends. Furthermore, the social inequality in frailty in men but not in women supports interventions specific to gender inequality and frailty.

## Figures and Tables

**Table 1:** Baseline characteristics of the study population according to subjective social status (community status ladder) at baseline and frailty status at 14-year follow-up (n = 694).

	Subjective Social Status					Frailty Status at Year 14		
	High (n = 414)	Middle (n = 228)	Low (n = 52)		Robust (n = 64)	Pre-Frail (n = 419)	Frail (n = 211)	
Variables	Mean ± SD/n (%)	Mean ± SD/n (%)	Mean ± SD/n (%)	*P*	Mean ± SD/n (%)	Mean ± SD/n (%)	Mean ± SD/n (%)	*P*
*Demographics*								
Age, years	69.7 ± 3.5	69.7 ± 3.7	69.2 ± 3.3	0.551	67.2 ± 2.2	69.3 ± 3.2	71.2 ± 3.9	<0.001
Sex								
Men	182 (44.0)	127 (55.7)	37 (71.2)	<0.001	38 (59.4)	230 (54.9)	78 (37.0)	<0.001
Women	232 (56.0)	101 (44.3)	15 (28.8)		26 (40.6)	189 (45.1)	133 (63.0)	
Marital status								
Married	322 (77.8)	186 (81.6)	47 (90.4)	0.077	58 (90.6)	343 (81.9)	154 (73.0)	0.003
Non-married (single, divorced, separated)	92 (22.2)	42 (18.4)	5 (9.6)		6 (9.4)	76 (18.1)	57 (27.0)	
*Objective socioeconomic status*								
Educational level								
At least completed primary	232 (56.0)	120 (52.6)	26 (50.0)	0.119	40 (62.5)	248 (59.2)	90 (42.7)	0.001
Some primary education	112 (27.1)	78 (34.2)	21 (40.4)		16 (25.0)	120 (28.6)	75 (35.5)	
No education	70 (16.9)	30 (13.2)	5 (9.6)		8 (12.5)	51 (12.2)	46 (21.8)	
Maximum life-time income								
Quartile 4 ≥HKD$14,000	97 (29.0)	40 (21.5)	12 (26.7)	0.328	19 (35.2)	101 (29.3)	29 (17.4)	<0.001
Quartile 3 HKD$8000–13,999	79 (23.6)	61 (32.8)	13 (28.9)		20 (37.0)	95 (27.5)	38 (22.8)	
Quartile 2 HKD$2500–7900	77 (23.0)	45 (24.2)	10 (22.2)		11 (20.4)	72 (20.9)	49 (29.3)	
Quartile 1 HKD$0–2499	82 (24.5)	40 (21.5)	10 (22.2)		4 (7.4)	77 (22.3)	51 (30.5)	
*Medical history*								
Hypertension								
No	251 (60.6)	137 (60.1)	31 (59.6)	0.984	48 (75.0)	267 (63.7)	104 (49.3)	<0.001
Yes	163 (39.4)	91 (39.9)	21 (40.4)		16 (25.0)	152 (36.3)	107 (50.7)	
Diabetes								
No	357 (86.2)	200 (87.7)	42 (80.8)	0.420	59 (92.2)	370 (88.3)	170 (80.6)	0.01
Yes	57 (13.8)	28 (12.3)	10 (19.2)		5 (7.8)	49 (11.7)	41 (19.4)	
Stroke								
No	399 (96.4)	226 (99.1)	49 (94.2)	0.060	63 (98.4)	411 (98.1)	200 (94.8)	0.052
Yes	15 (3.6)	2 (0.9)	3 (5.8)		1 (1.6)	8 (1.9)	11 (5.2)	
*Lifestyle*								
Current smoker								
No	397 (95.9)	222 (97.4)	50 (96.2)	0.628	61 (95.3)	403 (96.2)	205 (97.2)	0.732
Yes	17 (4.1)	6 (2.6)	2 (3.8)		3 (4.7)	16 (3.8)	6 (2.8)	
Current drinker								
No	199 (48.1)	91 (39.9)	18 (34.6)	0.047	23 (35.9)	184 (43.9)	101 (47.9)	0.232
Yes	215 (51.9)	137 (60.1)	34 (65.4)		41 (64.1)	235 (56.1)	110 (52.1)	
Physical activity, PASE score	106.7 ± 39.8	114.6 ± 47.7	115.7 ± 47.9	0.052	116.1 ± 42.0	112.0 ± 44.3	104.2 ± 41.3	0.052
Diet quality, DQI	66.0 ± 9.2	65.9 ± 9.0	66.5 ± 10.0	0.904	66.2 ± 8.7	65.7 ± 9.3	66.5 ± 9.1	0.651
BMI, kg/m^2^	24.0 ± 2.8	23.8 ± 2.8	24.0 ± 2.7	0.746	23.9 ± 2.4	23.6 ± 2.5	24.6 ± 3.3	<0.001
*Mental health*								
SF-12 MCS score	56.7 ± 5.8	56.9 ± 5.8	55.5 ± 5.8	0.286	56.1 ± 6.0	56.7 ± 5.9	56.7 ± 5.6	0.744
*cognitive function*								
MMSE score	26.8 ± 3.0	26.9 ± 2.8	27.7 ± 2.2	0.130	27.2 ± 2.9	27.1 ± 2.8	26.4 ± 3.1	0.010

Missing data: Maximum life-time income (Subjective social status: High, n = 79; Middle, n = 42; Low, n = 7; Frailty: Robust, n = 10; Pre-frail, n = 74; Frail, n = 44). PASE, Physical Activity Scale of the Elderly; DQI, Diet Quality Index; BMI, Body Mass Index; SF-12 MCS, Short Form-12 Mental Component Summary; MMSE, Mini-Mental Status Examination.

**Table 2:** Association between subjective social status (community status ladder), educational level, and maximum life-time income by sex (n = 694).

Variables	Men (n = 346)				Women (n = 348)		
	Subjective Social Status				Subjective Social Status		
	High (n = 182)	Middle (n = 127)	Low (n = 37)		High (n = 232)	Low to Middle (n = 116)	
	n (%)			*P*	n (%)		*P*
Educational level							
At least completed primary	139 (76.4)	90 (70.9)	20 (54.1)	0.064	93 (40.1)	30 (25.9)	0.132
Some primary	36 (19.8)	34 (26.8)	15 (40.5)		76 (32.8)	50 (43.1)	
No education	7 (3.8)	3 (2.4)	2 (5.4)		63 (27.2)	36 (31.0)	
Maximum life-time income							
Quartile 4 ≥HKD$14,000	78 (47.6)	39 (34.2)	12 (37.5)	0.240	44 (25.7)	16 (18.8)	0.405
Quartile 3 HKD$8000–13,999	54 (32.9)	47 (41.2)	12 (37.5)				
Quartile 2 HKD$2500–7900	25 (15.2)	20 (17.5)	4 (12.5)		52 (30.4)	31 (36.5)	
Quartile 1 HKD$0–2499	7 (4.3)	8 (7.0)	4 (12.5)		75 (43.9)	38 (44.7)	

*P*-values are based on chi-squared test. Subjective social status of low and middle groups for women were combined due to small sample sizes; Maximum life-time income of quartile 4 ≥HKD$14,000 and quartile 3 HKD$8000–13,999 for women were combined due to small sample sizes.

**Table 3:** Subjective social status (community status ladder) at baseline and the risk of incident frailty at 14-year follow-up, both sexes (n = 694).

	Model 1	Model 2	Model 3	Model 4	Model 5	Model 6	Model 7
Variables	OR (95% CI)	OR (95% CI)	OR (95% CI)	OR (95% CI)	OR (95% CI)	OR (95% CI)	OR (95% CI)
*Socioeconomic status*							
Subjective social status (ref = High)							
Middle	1.69 (1.21–2.38)	1.69 (1.21–2.38)	1.83 (1.24–2.68)	1.97 (1.33–2.92)	2.03 (1.36–3.02)	2.03 (1.36–3.01)	2.03 (1.36–3.02)
Low	2.43 (1.33–4.42)	2.44 (1.34–4.44)	2.43 (1.26–4.68)	2.29 (1.17–4.47)	2.30 (1.17–4.49)	2.35 (1.20–4.61)	2.34 (1.19–4.60)
*Socio-demographics*							
Age	1.22 (1.17–1.28)	1.22 (1.16–1.28)	1.22 (1.15–1.29)	1.22 (1.15–1.29)	1.22 (1.15–1.29)	1.22 (1.15–1.29)	1.22 (1.15–1.29)
Female (ref = Male)	2.15 (1.56–2.96)	2.08 (1.48–2.92)	2.04 (1.29–3.24)	2.01 (1.25–3.21)	2.15 (1.29–3.57)	2.16 (1.30–3.60)	2.17 (1.30–3.62)
Non-married (single, divorced, separated) (ref = Married)		1.12 (0.74–1.70)	0.95 (0.58–1.56)	0.88 (0.53–1.46)	0.89 (0.54–1.48)	0.90 (0.54–1.50)	0.90 (0.54–1.50)
Educational level (ref = At least completed primary)							
Some primary			1.08 (0.71–1.64)	1.06 (0.69–1.61)	1.02 (0.66–1.56)	1.00 (0.65–1.54)	1.01 (0.65–1.56)
No education			1.25 (0.70–2.25)	1.48 (0.81–2.71)	1.31 (0.71–2.43)	1.30 (0.70–2.41)	1.34 (0.68–2.62)
Maximum life-time income (ref = Quartile 4 ≥HKD$14,000)							
Quartile 3 HKD$8000–13,999			0.82 (0.50–1.34)	0.82 (0.50–1.35)	0.83 (0.50–1.37)	0.82 (0.50–1.36)	0.82 (0.50–1.36)
Quartile 2 HKD$2500–7900			1.17 (0.67–2.04)	1.25 (0.71–2.19)	1.30 (0.74–2.30)	1.32 (0.75–2.33)	1.33 (0.75–2.35)
Quartile 1 HKD$0–2499			1.12 (0.61–2.05)	1.15 (0.62–2.12)	1.19 (0.64–2.20)	1.18 (0.64–2.19)	1.19 (0.64–2.21)
*Medical history*							
Hypertension (ref = No hypertension)				1.93 (1.31–2.84)	1.83 (1.23–2.72)	1.83 (1.23–2.73)	1.84 (1.23–2.74)
Diabetes (ref = No diabetes)				1.86 (1.10–3.14)	1.78 (1.05–3.03)	1.78 (1.05–3.03)	1.78 (1.05–3.03)
Stroke (ref = No stroke)				3.57 (1.27–10.05)	3.79 (1.32–10.84)	3.74 (1.31–10.68)	3.75 (1.31–10.69)
*Lifestyle*							
Current smoker (ref = Non-current smoker)					1.19 (0.47–3.03)	1.18 (0.47–3.01)	1.18 (0.46–3.01)
Current drinker (ref = Non-current drinker)					1.11 (0.74–1.64)	1.10 (0.74–1.64)	1.10 (0.74–1.64)
Physical activity, PASE score					1.00 (1.00–1.00)	1.00 (1.00–1.00)	1.00 (1.00–1.00)
Diet quality, DQI					1.00 (0.98–1.01)	0.99 (0.98–1.01)	0.99 (0.98–1.01)
BMI, kg/m^2^					1.07 (1.01–1.15)	1.07 (1.00–1.15)	1.07 (1.00–1.15)
*Mental health*							
SF-12 MCS score						1.01 (0.98–1.05)	1.01 (0.98–1.05)
*Cognitive function*							
MMSE score							1.01 (0.94–1.08)

Model 1 was adjusted for age and sex at baseline; Model 2 was adjusted for variables in model1 plus marital status at baseline; Model 3 was adjusted for variables in model 2 plus educational level and maximum life-time income at baseline; Model 4 was adjusted for variables in model 3 plus medical history (hypertension, diabetes, and stroke) at baseline; Model 5 was adjusted for variables in model 4 plus lifestyle (smoking status, alcohol consumption, physical activity (PASE score), diet quality (DQI), BMI) at baseline; Model 6 was adjusted for variables in model 5 plus mental health (SF-12 MCS score) at baseline; Model 7 was adjusted for variables in model 6 plus cognitive function (MMSE score) at baseline; Ref, Reference; PASE, Physical Activity Scale of the Elderly; DQI, Diet Quality Index; BMI, Body Mass Index; SF-12 MCS, Short Form-12 Mental Component Summary; MMSE, Mini-Mental Status Examination.

**Table 4:** Subjective social status (community status ladder) at baseline and the risk of incident frailty at 14-year follow-up, men (n = 346).

	Model 1	Model 2	Model 3	Model 4	Model 5	Model 6	Model 7
Variables	OR (95% CI)	OR (95% CI)	OR (95% CI)	OR (95% CI)	OR (95% CI)	OR (95% CI)	OR (95% CI)
*Socioeconomic status*							
Subjective social status (ref = High)							
Middle	2.15 (1.31–3.53)	2.14 (1.31–3.52)	2.06 (1.22–3.49)	2.21 (1.29–3.78)	2.23 (1.30–3.83)	2.22 (1.29–3.82)	2.21 (1.28–3.81)
Low	3.70 (1.74–7.88)	3.67 (1.73–7.82)	3.75 (1.66–8.47)	3.44 (1.48–7.97)	3.42 (1.47–7.97)	3.45 (1.48–8.07)	3.60 (1.54–8.41)
*Socio–demographics*							
Age	1.23 (1.15–1.32)	1.23 (1.15–1.32)	1.18 (1.09–1.28)	1.19 (1.10–1.29)	1.20 (1.11–1.30)	1.20 (1.11–1.30)	1.20 (1.11–1.30)
Non–married (single, divorced, separated) (ref = Married)		0.73 (0.28–1.95)	0.70 (0.24–2.01)	0.58 (0.20–1.70)	0.60 (0.20–1.77)	0.61 (0.21–1.81)	0.62 (0.21–1.86)
Educational level (ref = At least completed primary)							
Some primary			1.01 (0.57–1.79)	0.95 (0.53–1.71)	0.93 (0.51–1.68)	0.89 (0.49–1.62)	0.81 (0.44–1.51)
No education			2.11 (0.54–8.16)	3.05 (0.75–12.43)	2.80 (0.66–11.93)	2.89 (0.68–12.36)	2.30 (0.52–10.09)
Maximum life–time income (ref = Quartile 4 ≥HKD$14,000)							
Quartile 3 HKD$8000–13,999			1.21 (0.69–2.14)	1.21 (0.68–2.16)	1.23 (0.69–2.20)	1.22 (0.68–2.19)	1.24 (0.69–2.22)
Quartile 2 HKD$2500–7900			1.37 (0.66–2.84)	1.39 (0.66–2.92)	1.45 (0.68–3.08)	1.47 (0.69–3.11)	1.37 (0.64–2.93)
Quartile 1 HKD$0–2499			1.43 (0.51–4.05)	1.50 (0.52–4.30)	1.43 (0.50–4.15)	1.48 (0.51–4.29)	1.35 (0.46–3.97)
*Medical history*							
Hypertension (ref = No hypertension)				1.93 (1.12–3.33)	1.80 (1.02–3.18)	1.82 (1.03–3.21)	1.74 (0.98–3.09)
Diabetes (ref = No diabetes)				2.02 (0.97–4.24)	1.95 (0.93–4.12)	2.01 (0.95–4.27)	1.99 (0.93–4.23)
Stroke (ref = No stroke)				3.07 (0.86–10.99)	3.12 (0.86–11.40)	3.15 (0.87–11.48)	3.28 (0.90–12.00)
*Lifestyle*							
Current smoker (ref = Non–current smoker)					1.18 (0.42–3.30)	1.15 (0.41–3.24)	1.13 (0.40–3.19)
Current drinker (ref = Non–current drinker)					1.00 (0.56–1.79)	1.02 (0.57–1.83)	1.03 (0.57–1.86)
Physical activity, PASE score					1.00 (1.00–1.01)	1.00 (1.00–1.01)	1.00 (1.00–1.01)
Diet quality, DQI					1.00 (0.97–1.03)	1.00 (0.97–1.02)	1.00 (0.97–1.02)
BMI, kg/m^2^					1.07 (0.97–1.18)	1.07 (0.97–1.18)	1.07 (0.97–1.18)
*Mental health*							
SF–12 MCS score						1.02 (0.97–1.07)	1.02 (0.98–1.07)
*Cognitive function*							
MMSE score							0.92 (0.81–1.05)

Model 1 was adjusted for age at baseline; Model 2 was adjusted for variable in model 1 plus marital status at baseline; Model 3 was adjusted for variables in model 2 plus educational level and maximum life–time income at baseline; Model 4 was adjusted for variables in model 3 plus medical history (hypertension, diabetes, and stroke) at baseline; Model 5 was adjusted for variables in model 4 plus lifestyle (smoking status, alcohol consumption, physical activity (PASE score), diet quality (DQI), BMI) at baseline; Model 6 was adjusted for variables in model 5 plus mental health (SF-12 MCS score) at baseline; Model 7 was adjusted for variables in model 6 plus cognitive function (MMSE score) at baseline; Ref, Reference; PASE, Physical Activity Scale of the Elderly; DQI, Diet Quality Index; BMI, Body Mass Index; SF-12 MCS, Short Form-12 Mental Component Summary; MMSE, Mini-Mental Status Examination.

**Table 5:** Subjective social status (community status ladder) at baseline and the risk of incident frailty at 14-year follow-up, women (n = 348).

	Model 1	Model 2	Model 3	Model 4	Model 5	Model 6	Model 7
Variables	OR (95% CI)	OR (95% CI)	OR (95% CI)	OR (95% CI)	OR (95% CI)	OR (95% CI)	OR (95% CI)
*Socioeconomic status*							
Subjective social status (ref = High)							
Low to middle *	1.37 (0.87–2.16)	1.37 (0.87–2.16)	1.45 (0.84–2.51)	1.56 (0.89–2.72)	1.60 (0.91–2.82)	1.61 (0.91–2.83)	1.61 (0.91–2.84)
*Socio–demographics*							
Age	1.22 (1.14–1.29)	1.21 (1.13–1.29)	1.26 (1.16–1.36)	1.25 (1.15–1.35)	1.24 (1.14–1.35)	1.24 (1.14–1.35)	1.25 (1.14–1.36)
Non–married (single, divorced, separated) (ref = Married)		1.22 (0.77–1.93)	1.01 (0.57–1.79)	0.96 (0.54–1.71)	0.98 (0.55–1.75)	0.98 (0.55–1.75)	0.97 (0.54–1.74)
Educational level (ref = At least completed primary)							
Some primary			0.97 (0.52–1.82)	1.01 (0.53–1.91)	0.95 (0.50–1.83)	0.95 (0.50–1.83)	1.00 (0.52–1.94)
No education			0.95 (0.47–1.91)	1.15 (0.56–2.37)	1.03 (0.49–2.15)	1.02 (0.49–2.15)	1.26 (0.55–2.89)
Maximum life–time income (ref = Quartile 3 and 4 (HKD$8000–13,999 and ≥HKD$14,000 ^))							
Quartile 2 HKD$2500–7900			1.64 (0.79–3.41)	1.90 (0.90–3.99)	1.89 (0.88–4.06)	1.91 (0.89–4.13)	1.98 (0.91–4.29)
Quartile 1 HKD$0–2499			1.46 (0.73–2.92)	1.56 (0.77–3.15)	1.57 (0.76–3.25)	1.57 (0.76–3.25)	1.58 (0.77–3.28)
*Medical history*							
Hypertension (ref = No hypertension)				2.08 (1.19–3.65)	2.00 (1.12–3.58)	1.99 (1.11–3.57)	2.03 (1.13–3.64)
Diabetes (ref = No diabetes)				1.60 (0.73–3.48)	1.50 (0.68–3.30)	1.49 (0.67–3.27)	1.45 (0.66–3.21)
Stroke (ref = No stroke)				5.70 (0.81–40.04)	6.67 (0.93–47.74)	6.44 (0.89–46.55)	6.72 (0.93–48.44)
*Lifestyle*							
Current smoker (ref = Non–current smoker)					0.62 (0.05–8.10)	0.62 (0.05–8.07)	0.52 (0.04–6.64)
Current drinker (ref = Non–current drinker)					1.22 (0.70–2.15)	1.21 (0.69–2.13)	1.24 (0.70–2.19)
Physical activity, PASE score					1.00 (0.99–1.01)	1.00 (0.99–1.01)	1.00 (0.99–1.01)
Diet quality, DQI					0.99 (0.97–1.02)	0.99 (0.97–1.02)	0.99 (0.96–1.02)
BMI, kg/m^2^					1.08 (0.98–1.19)	1.08 (0.98–1.18)	1.08 (0.98–1.19)
*Mental health*							
SF–12 MCS score						1.01 (0.97–1.05)	1.01 (0.97–1.06)
*Cognitive function*							
MMSE score							1.06 (0.96–1.16)

* Subjective social status of low and middle groups were combined due to small sample sizes; ^ Maximum life–time income of quartile 3 and 4 (HKD$8000–13,999 and ≥HKD$14,000) were combined due to small sample sizes; Model 1 was adjusted for age at baseline; Model 2 was adjusted for variable in model 1 plus marital status at baseline; Model 3 was adjusted for variables in model 2 plus educational level and maximum life-time income at baseline; Model 4 was adjusted for variables in model 3 plus medical history (hypertension, diabetes, and stroke) at baseline; Model 5 was adjusted for variables in model 4 plus lifestyle (smoking status, alcohol consumption, physical activity (PASE score), diet quality (DQI), BMI) at baseline; Model 6 was adjusted for variables in model 5 plus mental health (SF-12 MCS score) at baseline; Model 7 was adjusted for variables in model 6 plus cognitive function (MMSE score) at baseline; Ref, reference; PASE, Physical Activity Scale of the Elderly; DQI, Diet Quality Index; BMI, Body Mass Index; SF-12 MCS, Short Form-12 Mental Component Summary; MMSE, Mini-Mental Status Examination.

## Supplementary Materials

The following are available online at https://www.mdpi.com/1660-4601/17/4/1301/s1, Table S1a Educational level at baseline and the risk of incident frailty at 14-year follow-up, both sexes (n = 694), Table S1b Educational level at baseline and the risk of incident frailty at 14-year follow-up, men (n = 346), Table S1c Educational level at baseline and the risk of incident frailty at 14-year follow-up, women (n = 348), Table S2a Maximum life-time income and the risk of incident frailty at 14-year follow-up, both sexes (n = 566), Table S2b Maximum life-time income and the risk of incident frailty at 14-year follow-up, men (n = 310), Table S2c Maximum life-time income and the risk of incident frailty at 14-year follow-up, women (n = 256), Table S3 Baseline characteristics of the study population retained in the 14-year follow-up versus those loss to follow-up (n = 4000).
