# Supplementary material for: Socioeconomic Inequalities in Frailty in Hong Kong, China: A 14-Year Longitudinal Cohort Study
Source: Int J Environ Res Public Health. 2020 Feb 18;17(4):1301. doi: 10.3390/ijerph17041301 (PMC7068288; doi:10.3390/ijerph17041301)
Supplement: Supplementary file 1 [file ijerph-17-01301-s001.pdf]

Table S1a Educational level at baseline and the risk of incident frailty at 14-year follow-up, both sexes (n=694)

	Model 1	Model 2	Model 3	Model 4	Model 5	Model 6	Model 7	Model 8
Variables	OR (95% CI)	OR (95% CI)	OR (95% CI)	OR (95% CI)	OR (95% CI)	OR (95% CI)	OR (95% CI)	OR (95% CI)
Socio-demographics								
Educational level (ref=At least completed primary)								
Some primary	1.22 (0.85-1.76)	1.22 (0.85-1.76)	1.17 (0.77-1.77)	1.08 (0.71-1.64)	1.06 (0.69-1.61)	1.02 (0.66-1.56)	1.00 (0.65-1.54)	1.01 (0.65-1.56)
No education	1.34 (0.82-2.18)	1.32 (0.80-2.16)	1.27 (0.71-2.27)	1.25 (0.70-2.25)	1.48 (0.81-2.71)	1.31 (0.71-2.43)	1.30 (0.70-2.41)	1.34 (0.68-2.62)
Age	1.21 (1.15-1.27)	1.21 (1.15-1.27)	1.21 (1.15-1.28)	1.22 (1.15-1.29)	1.22 (1.15-1.29)	1.22 (1.15-1.29)	1.22 (1.15-1.29)	1.22 (1.15-1.29)
Female (ref=Male)	1.77 (1.26-2.49)	1.74 (1.23-2.48)	1.77 (1.13-2.78)	2.04 (1.29-3.24)	2.01 (1.25-3.21)	2.15 (1.29-3.57)	2.16 (1.30-3.60)	2.17 (1.30-3.62)
Non-married (single, divorced, separated) (ref=Married)		1.07 (0.71-1.63)	0.93 (0.57-1.53)	0.95 (0.58-1.56)	0.88 (0.53-1.46)	0.89 (0.54-1.48)	0.90 (0.54-1.50)	0.90 (0.54-1.50)
Maximum life-time income (ref= Quartile 4 ≥HKD$14,000)								
Quartile 3 HKD$8,000-13,999			0.89 (0.55-1.45)	0.82 (0.50-1.34)	0.82 (0.50-1.35)	0.83 (0.50-1.37)	0.82 (0.50-1.36)	0.82 (0.50-1.36)
Quartile 2 HKD$2,500-7900			1.29 (0.75-2.24)	1.17 (0.67-2.04)	1.25 (0.71-2.19)	1.30 (0.74-2.30)	1.32 (0.75-2.33)	1.33 (0.75-2.35)
Quartile 1 HKD$0-2,499			1.22 (0.67-2.23)	1.12 (0.61-2.05)	1.15 (0.62-2.12)	1.19 (0.64-2.20)	1.18 (0.64-2.19)	1.19 (0.64-2.21)
Socioeconomic status								
Subjective social status (ref=High)								
Middle				1.83 (1.24-2.68)	1.97 (1.33-2.92)	2.03 (1.36-3.02)	2.03 (1.36-3.01)	2.03 (1.36-3.02)
Low				2.43 (1.26-4.68)	2.29 (1.17-4.47)	2.30 (1.17-4.49)	2.35 (1.20-4.61)	2.34 (1.19-4.60)
Medical history								
Hypertension (ref=No hypertension)					1.93 (1.31-2.84)	1.83 (1.23-2.72)	1.83 (1.23-2.73)	1.84 (1.23-2.74)
Diabetes (ref=No diabetes)					1.86 (1.10-3.14)	1.78 (1.05-3.03)	1.78 (1.05-3.03)	1.78 (1.05-3.03)
Stroke (ref=No stroke)					3.57 (1.27-10.05)	3.79 (1.32-10.84)	3.74 (1.31-10.68)	3.75 (1.31-10.69)
Lifestyle								
Current smoker (ref=Non-current						1.19 (0.47-3.03)	1.18 (0.47-3.01)	1.18 (0.46-3.01)

smoker)			
Current drinker (ref=Non-current drinker)	1.11 (0.74-1.64)	1.10 (0.74-1.64)	1.10 (0.74-1.64)
Physical activity, PASE score	1.00 (1.00-1.00)	1.00 (1.00-1.00)	1.00 (1.00-1.00)
Diet quality, DQI	1.00 (0.98-1.01)	0.99 (0.98-1.01)	0.99 (0.98-1.01)
BMI, kg/m^2	1.07 (1.01-1.15)	1.07 (1.00-1.15)	1.07 (1.00-1.15)
Mental health			
SF-12 MCS score		1.01 (0.98-1.05)	1.01 (0.98-1.05)
Cognitive function			
MMSE score			1.01 (0.94-1.08)

Model 1 was adjusted for age and sex at baseline
Model 2 was adjusted for variables in model 1 plus marital status at baseline
Model 3 was adjusted for variables in model 2 plus maximum life-time income at baseline
Model 4 was adjusted for variables in model 3 plus subjective social status (community status ladder) at baseline
Model 5 was adjusted for variables in model 4 plus medical history (hypertension, diabetes, and stroke) at baseline
Model 6 was adjusted for variables in model 5 plus lifestyle (smoking status, alcohol consumption, physical activity (PASE score), diet quality (DQI), BMI) at baseline
Model 7 was adjusted for variables in model 6 plus mental health (SF-12 MCS) at baseline
Model 8 was adjusted for variables in model 7 plus cognitive function (MMSE) at baseline
Ref, Reference; PASE, Physical Activity Scale of the Elderly; DQI, Diet Quality Index; BMI, Body Mass Index; SF-12 MCS, Short Form-12 Mental Component Summary; MMSE, Mini-Mental Status Examination

Table S1b Educational level at baseline and the risk of incident frailty at 14-year follow-up, men (n=346)

	Model 1	Model 2	Model 3	Model 4	Model 5	Model 6	Model 7	Model 8
Variables	OR (95% CI)	OR (95% CI)	OR (95% CI)	OR (95% CI)	OR (95% CI)	OR (95% CI)	OR (95% CI)	OR (95% CI)
Socio-demographics								
Educational level (ref=At least completed primary)								
Some primary	1.39 (0.82-2.36)	1.36 (0.80-2.32)	1.16 (0.66-2.03)	1.01 (0.57-1.79)	0.95 (0.53-1.71)	0.93 (0.51-1.68)	0.89 (0.49-1.62)	0.81 (0.44-1.51)
No education	1.77 (0.51-6.23)	1.85 (0.52-6.55)	2.25 (0.62-8.25)	2.11 (0.54-8.16)	3.05 (0.75-12.43)	2.80 (0.66-11.93)	2.89 (0.68-12.36)	2.30 (0.52-10.09)
Age	1.21 (1.13-1.30)	1.22 (1.13-1.31)	1.17 (1.09-1.26)	1.18 (1.09-1.28)	1.19 (1.10-1.29)	1.20 (1.11-1.30)	1.20 (1.11-1.30)	1.20 (1.11-1.30)
Non-married (single, divorced, separated) (ref=Married)		0.71 (0.27-1.88)	0.71 (0.25-2.01)	0.70 (0.24-2.01)	0.58 (0.20-1.70)	0.60 (0.20-1.77)	0.61 (0.21-1.81)	0.62 (0.21-1.86)
Maximum life-time income (ref= Quartile 4 ≥HKD$14,000)								
Quartile 3 HKD$8,000-13,999			1.31 (0.75-2.30)	1.21 (0.69-2.14)	1.21 (0.68-2.16)	1.23 (0.69-2.20)	1.22 (0.68-2.19)	1.24 (0.69-2.22)
Quartile 2 HKD$2,500-7900			1.41 (0.69-2.87)	1.37 (0.66-2.84)	1.39 (0.66-2.92)	1.45 (0.68-3.08)	1.47 (0.69-3.11)	1.37 (0.64-2.93)
Quartile 1 HKD$0-2,499			1.72 (0.62-4.80)	1.43 (0.51-4.05)	1.50 (0.52-4.30)	1.43 (0.50-4.15)	1.48 (0.51-4.29)	1.35 (0.46-3.97)
Socioeconomic status								
Subjective social status (ref=High)								
Middle				2.06 (1.22-3.49)	2.21 (1.29-3.78)	2.23 (1.30-3.83)	2.22 (1.29-3.82)	2.21 (1.28-3.81)
Low				3.75 (1.66-8.47)	3.44 (1.48-7.97)	3.42 (1.47-7.97)	3.45 (1.48-8.07)	3.60 (1.54-8.41)
Medical history								
Hypertension (ref=No hypertension)					1.93 (1.12-3.33)	1.80 (1.02-3.18)	1.82 (1.03-3.21)	1.74 (0.98-3.09)
Diabetes (ref=No diabetes)					2.02 (0.97-4.24)	1.95 (0.93-4.12)	2.01 (0.95-4.27)	1.99 (0.93-4.23)
Stroke (ref=No stroke)					3.07 (0.86-10.99)	3.12 (0.86-11.40)	3.15 (0.87-11.48)	3.28 (0.90-12.00)
Lifestyle								
Current smoker (ref=Non-current						1.18 (0.42-3.30)	1.15 (0.41-3.24)	1.13 (0.40-3.19)

smoker)			
Current drinker (ref=Non-current drinker)	1.00 (0.56-1.79)	1.02 (0.57-1.83)	1.03 (0.57-1.86)
Physical activity, PASE score	1.00 (1.00-1.01)	1.00 (1.00-1.01)	1.00 (1.00-1.01)
Diet quality, DQI	1.00 (0.97-1.03)	1.00 (0.97-1.02)	1.00 (0.97-1.02)
BMI, kg/m^2	1.07 (0.97-1.18)	1.07 (0.97-1.18)	1.07 (0.97-1.18)
Mental health			
SF-12 MCS score		1.02 (0.97-1.07)	1.02 (0.98-1.07)
Cognitive function			
MMSE score			0.92 (0.81-1.05)

Model 1 was adjusted for age at baseline
Model 2 was adjusted for variable in model 1 plus marital status at baseline
Model 3 was adjusted for variables in model 2 plus maximum life-time income at baseline
Model 4 was adjusted for variables in model 3 plus subjective social status (community status ladder) at baseline
Model 5 was adjusted for variables in model 4 plus medical history (hypertension, diabetes, and stroke) at baseline
Model 6 was adjusted for variables in model 5 plus lifestyle (smoking status, alcohol consumption, physical activity (PASE score), diet quality (DQI), BMI) at baseline
Model 7 was adjusted for variables in model 6 plus mental health (SF-12 MCS) at baseline
Model 8 was adjusted for variables in model 7 plus cognitive function (MMSE) at baseline
Ref, Reference; PASE, Physical Activity Scale of the Elderly; DQI, Diet Quality Index; BMI, Body Mass Index; SF-12 MCS, Short Form-12 Mental Component Summary; MMSE, Mini-Mental Status Examination

Table S1c Educational level at baseline and the risk of incident frailty at 14-year follow-up, women (n=348)

	Model 1	Model 2	Model 3	Model 4	Model 5	Model 6	Model 7	Model 8
Variables	OR (95% CI)	OR (95% CI)	OR (95% CI)	OR (95% CI)	OR (95% CI)	OR (95% CI)	OR (95% CI)	OR (95% CI)
Socio-demographics								
Educational level (ref=At least completed primary)								
Some primary	1.08 (0.66-1.79)	1.07 (0.65-1.77)	1.02 (0.55-1.91)	0.97 (0.52-1.82)	1.01 (0.53-1.91)	0.95 (0.50-1.83)	0.95 (0.50-1.83)	1.00 (0.52-1.94)
No education	1.20 (0.69-2.08)	1.15 (0.66-2.02)	0.96 (0.48-1.92)	0.95 (0.47-1.91)	1.15 (0.56-2.37)	1.03 (0.49-2.15)	1.02 (0.49-2.15)	1.26 (0.55-2.89)
Age	1.21 (1.13-1.29)	1.20 (1.13-1.28)	1.25 (1.16-1.36)	1.26 (1.16-1.36)	1.25 (1.15-1.35)	1.24 (1.14-1.35)	1.24 (1.14-1.35)	1.25 (1.14-1.36)
Non-married (single, divorced, separated) (ref=Married)		1.19 (0.75-1.90)	0.99 (0.56-1.75)	1.01 (0.57-1.79)	0.96 (0.54-1.71)	0.98 (0.55-1.75)	0.98 (0.55-1.75)	0.97 (0.54-1.74)
Maximum life-time income (ref= Quartile 3 and 4 (HKD$8,000-13,999 and ≥HKD$14,000^))								
Quartile 2 HKD$2,500-7900			1.73 (0.84-3.58)	1.64 (0.79-3.41)	1.90 (0.90-3.99)	1.89 (0.88-4.06)	1.91 (0.89-4.13)	1.98 (0.91-4.29)
Quartile 1 HKD$0-2,499			1.50 (0.75-3.00)	1.46 (0.73-2.92)	1.56 (0.77-3.15)	1.57 (0.76-3.25)	1.57 (0.76-3.25)	1.58 (0.77-3.28)
Socioeconomic status								
Subjective social status (ref=High)								
Low to middle*				1.45 (0.84-2.51)	1.56 (0.89-2.72)	1.60 (0.91-2.82)	1.61 (0.91-2.83)	1.61 (0.91-2.84)
Medical history								
Hypertension (ref=No hypertension)					2.08 (1.19-3.65)	2.00 (1.12-3.58)	1.99 (1.11-3.57)	2.03 (1.13-3.64)
Diabetes (ref=No diabetes)					1.60 (0.73-3.48)	1.50 (0.68-3.30)	1.49 (0.67-3.27)	1.45 (0.66-3.21)
Stroke (ref=No stroke)					5.70 (0.81-40.04)	6.67 (0.93-47.74)	6.44 (0.89-46.55)	6.72 (0.93-48.44)
Lifestyle								
Current smoker (ref=Non-current						0.62 (0.05-8.10)	0.62 (0.05-8.07)	0.52 (0.04-6.64)

smoker)			
Current drinker (ref=Non-current drinker)	1.22 (0.70-2.15)	1.21 (0.69-2.13)	1.24 (0.70-2.19)
Physical activity, PASE score	1.00 (0.99-1.01)	1.00 (0.99-1.01)	1.00 (0.99-1.01)
Diet quality, DQI	0.99 (0.97-1.02)	0.99 (0.97-1.02)	0.99 (0.96-1.02)
BMI, kg/m^2	1.08 (0.98-1.19)	1.08 (0.98-1.18)	1.08 (0.98-1.19)
Mental health			
SF-12 MCS score		1.01 (0.97-1.05)	1.01 (0.97-1.06)
Cognitive function			
MMSE score			1.06 (0.96-1.16)

^ Maximum life-time income of quartile 3 and 4 (HKD$8,000-13,999 and ≥HKD$14,000) were combined due to small sample sizes
* Subjective social status of low and middle groups were combined due to small sample sizes
Model 1 was adjusted for age at baseline
Model 2 was adjusted for variable in model 1 plus marital status at baseline
Model 3 was adjusted for variables in model 2 plus maximum life-time income at baseline
Model 4 was adjusted for variables in model 3 plus subjective social status (community status ladder) at baseline
Model 5 was adjusted for variables in model 4 plus medical history (hypertension, diabetes, and stroke) at baseline
Model 6 was adjusted for variables in model 5 plus lifestyle (smoking status, alcohol consumption, physical activity (PASE score), diet quality (DQI), BMI) at baseline
Model 7 was adjusted for variables in model 6 plus mental health (SF-12 MCS) at baseline
Model 8 was adjusted for variables in model 7 plus cognitive function (MMSE) at baseline
Ref, Reference; PASE, Physical Activity Scale of the Elderly; DQI, Diet Quality Index; BMI, Body Mass Index; SF-12 MCS, Short Form-12 Mental Component Summary; MMSE, Mini-Mental Status Examination

Table S2a Maximum life-time income and the risk of incident frailty at 14-year follow-up, both sexes (n=566)

	Model 1	Model 2	Model 3	Model 4	Model 5	Model 6	Model 7	Model 8
Variables	OR (95% CI)	OR (95% CI)	OR (95% CI)	OR (95% CI)	OR (95% CI)	OR (95% CI)	OR (95% CI)	OR (95% CI)
Socio-demographics								
Maximum life-time income (ref= Quartile 4 ≥HKD$14,000)								
Quartile 3 HKD$8,000-13,999	0.92 (0.57-1.49)	0.92 (0.57-1.49)	0.89 (0.55-1.45)	0.82 (0.50-1.34)	0.82 (0.50-1.35)	0.83 (0.50-1.37)	0.82 (0.50-1.36)	0.82 (0.50-1.36)
Quartile 2 HKD$2,500-7900	1.35 (0.79-2.31)	1.36 (0.79-2.32)	1.29 (0.75-2.24)	1.17 (0.67-2.04)	1.25 (0.71-2.19)	1.30 (0.74-2.30)	1.32 (0.75-2.33)	1.33 (0.75-2.35)
Quartile 1 HKD$0-2,499	1.31 (0.73-2.34)	1.31 (0.73-2.34)	1.22 (0.67-2.23)	1.12 (0.61-2.05)	1.15 (0.62-2.12)	1.19 (0.64-2.20)	1.18 (0.64-2.19)	1.19 (0.64-2.21)
Age	1.21 (1.15-1.28)	1.22 (1.15-1.28)	1.21 (1.15-1.28)	1.22 (1.15-1.29)	1.22 (1.15-1.29)	1.22 (1.15-1.29)	1.22 (1.15-1.29)	1.22 (1.15-1.29)
Female (ref=Male)	1.81 (1.18-2.76)	1.82 (1.17-2.84)	1.77 (1.13-2.78)	2.04 (1.29-3.24)	2.01 (1.25-3.21)	2.15 (1.29-3.57)	2.16 (1.30-3.60)	2.17 (1.30-3.62)
Non-married (single, divorced, separated) (ref=Married)		0.96 (0.60-1.56)	0.93 (0.57-1.53)	0.95 (0.58-1.56)	0.88 (0.53-1.46)	0.89 (0.54-1.48)	0.90 (0.54-1.50)	0.90 (0.54-1.50)
Educational level (ref=At least completed primary)								
Some primary			1.17 (0.77-1.77)	1.08 (0.71-1.64)	1.06 (0.69-1.61)	1.02 (0.66-1.56)	1.00 (0.65-1.54)	1.01 (0.65-1.56)
No education			1.27 (0.71-2.27)	1.25 (0.70-2.25)	1.48 (0.81-2.71)	1.31 (0.71-2.43)	1.30 (0.70-2.41)	1.34 (0.68-2.62)
Socioeconomic status								
Subjective social status (ref=High)								
Middle				1.83 (1.24-2.68)	1.97 (1.33-2.92)	2.03 (1.36-3.02)	2.03 (1.36-3.01)	2.03 (1.36-3.02)
Low				2.43 (1.26-4.68)	2.29 (1.17-4.47)	2.30 (1.17-4.49)	2.35 (1.20-4.61)	2.34 (1.19-4.60)
Medical history								
Hypertension (ref=No hypertension)					1.93 (1.31-2.84)	1.83 (1.23-2.72)	1.83 (1.23-2.73)	1.84 (1.23-2.74)
Diabetes (ref=No diabetes)					1.86 (1.10-3.14)	1.78 (1.05-3.03)	1.78 (1.05-3.03)	1.78 (1.05-3.03)
Stroke (ref=No stroke)					3.57 (1.27-10.05)	3.79 (1.32-10.84)	3.74 (1.31-10.68)	3.75 (1.31-10.69)
Lifestyle								

Current smoker (ref=Non-current smoker)	1.19 (0.47-3.03)	1.18 (0.47-3.01)	1.18 (0.46-3.01)
Current drinker (ref=Non-current drinker)	1.11 (0.74-1.64)	1.10 (0.74-1.64)	1.10 (0.74-1.64)
Physical activity, PASE score	1.00 (1.00-1.00)	1.00 (1.00-1.00)	1.00 (1.00-1.00)
Diet quality, DQI	1.00 (0.98-1.01)	0.99 (0.98-1.01)	0.99 (0.98-1.01)
BMI, kg/m^2	1.07 (1.01-1.15)	1.07 (1.00-1.15)	1.07 (1.00-1.15)
Mental health			
SF-12 MCS score		1.01 (0.98-1.05)	1.01 (0.98-1.05)
Cognitive function			
MMSE score			1.01 (0.94-1.08)

Model 1 was adjusted for age and sex at baseline
Model 2 was adjusted for variables in model 1 plus marital status at baseline
Model 3 was adjusted for variables in model 2 plus educational level at baseline
Model 4 was adjusted for variables in model 3 plus subjective social status (community status ladder) at baseline
Model 5 was adjusted for variables in model 4 plus medical history (hypertension, diabetes, and stroke) at baseline
Model 6 was adjusted for variables in model 5 plus lifestyle (smoking status, alcohol consumption, physical activity (PASE score), diet quality (DQI), BMI) at baseline
Model 7 was adjusted for variables in model 6 plus mental health (SF-12 MCS) at baseline
Model 8 was adjusted for variables in model 7 plus cognitive function (MMSE) at baseline
Ref, Reference; PASE, Physical Activity Scale of the Elderly; DQI, Diet Quality Index; BMI, Body Mass Index; SF-12 MCS, Short Form-12 Mental Component Summary; MMSE, Mini-Mental Status Examination

Table S2b Maximum life-time income and the risk of incident frailty at 14-year follow-up, men (n=310)

	Model 1	Model 2	Model 3	Model 4	Model 5	Model 6	Model 7	Model 8
Variables	OR (95% CI)	OR (95% CI)	OR (95% CI)	OR (95% CI)	OR (95% CI)	OR (95% CI)	OR (95% CI)	OR (95% CI)
Socio-demographics								
Maximum life-time income (ref= Quartile 4 ≥HKD$14,000)								
Quartile 3 HKD$8,000-13,999	1.36 (0.78-2.36)	1.37 (0.79-2.37)	1.31 (0.75-2.30)	1.21 (0.69-2.14)	1.21 (0.68-2.16)	1.23 (0.69-2.20)	1.22 (0.68-2.19)	1.24 (0.69-2.22)
Quartile 2 HKD$2,500-7900	1.39 (0.69-2.80)	1.42 (0.70-2.87)	1.41 (0.69-2.87)	1.37 (0.66-2.84)	1.39 (0.66-2.92)	1.45 (0.68-3.08)	1.47 (0.69-3.11)	1.37 (0.64-2.93)
Quartile 1 HKD$0-2,499	1.72 (0.62-4.76)	1.72 (0.62-4.77)	1.72 (0.62-4.80)	1.43 (0.51-4.05)	1.50 (0.52-4.30)	1.43 (0.50-4.15)	1.48 (0.51-4.29)	1.35 (0.46-3.97)
Age	1.17 (1.08-1.26)	1.17 (1.09-1.26)	1.17 (1.09-1.26)	1.18 (1.09-1.28)	1.19 (1.10-1.29)	1.20 (1.11-1.30)	1.20 (1.11-1.30)	1.20 (1.11-1.30)
Non-married (single, divorced, separated) (ref=Married)		0.75 (0.27-2.07)	0.71 (0.25-2.01)	0.70 (0.24-2.01)	0.58 (0.20-1.70)	0.60 (0.20-1.77)	0.61 (0.21-1.81)	0.62 (0.21-1.86)
Educational level (ref=At least completed primary)								
Some primary			1.16 (0.66-2.03)	1.01 (0.57-1.79)	0.95 (0.53-1.71)	0.93 (0.51-1.68)	0.89 (0.49-1.62)	0.81 (0.44-1.51)
No education			2.25 (0.62-8.25)	2.11 (0.54-8.16)	3.05 (0.75-12.43)	2.80 (0.66-11.93)	2.89 (0.68-12.36)	2.30 (0.52-10.09)
Subjective social status (ref=High)								
Middle				2.06 (1.22-3.49)	2.21 (1.29-3.78)	2.23 (1.30-3.83)	2.22 (1.29-3.82)	2.21 (1.28-3.81)
Low				3.75 (1.66-8.47)	3.44 (1.48-7.97)	3.42 (1.47-7.97)	3.45 (1.48-8.07)	3.60 (1.54-8.41)
Medical history								
Hypertension (ref=No hypertension)					1.93 (1.12-3.33)	1.80 (1.02-3.18)	1.82 (1.03-3.21)	1.74 (0.98-3.09)
Diabetes (ref=No diabetes)					2.02 (0.97-4.24)	1.95 (0.93-4.12)	2.01 (0.95-4.27)	1.99 (0.93-4.23)
Stroke (ref=No stroke)					3.07 (0.86-10.99)	3.12 (0.86-11.40)	3.15 (0.87-11.48)	3.28 (0.90-12.00)
Lifestyle								
Current smoker (ref=No current smoker)						1.18 (0.42-3.30)	1.15 (0.41-3.24)	1.13 (0.40-3.19)
Current drinker (ref=No current						1.00 (0.56-1.79)	1.02 (0.57-1.83)	1.03 (0.57-1.86)

drinker)			
Physical activity, PASE score	1.00 (1.00-1.01)	1.00 (1.00-1.01)	1.00 (1.00-1.01)
Diet quality, DQI	1.00 (0.97-1.03)	1.00 (0.97-1.02)	1.00 (0.97-1.02)
BMI, kg/m^2	1.07 (0.97-1.18)	1.07 (0.97-1.18)	1.07 (0.97-1.18)
Mental health			
SF-12 MCS score		1.02 (0.97-1.07)	1.02 (0.98-1.07)
Cognitive function			
MMSE score			0.92 (0.81-1.05)

Model 1 was adjusted for age at baseline
Model 2 was adjusted for variable in model 1 plus marital status at baseline
Model 3 was adjusted for variables in model 2 plus educational level at baseline
Model 4 was adjusted for variables in model 3 plus subjective social status (community status ladder) at baseline
Model 5 was adjusted for variables in model 4 plus medical history (hypertension, diabetes, and stroke) at baseline
Model 6 was adjusted for variables in model 5 plus lifestyle (smoking status, alcohol consumption, physical activity (PASE score), diet quality (DQI), BMI) at baseline
Model 7 was adjusted for variables in model 6 plus mental health (SF-12 MCS) at baseline
Model 8 was adjusted for variables in model 7 plus cognitive function (MMSE) at baseline
Ref, Reference; PASE, Physical Activity Scale of the Elderly; DQI, Diet Quality Index; BMI, Body Mass Index; SF-12 MCS, Short Form-12 Mental Component Summary; MMSE, Mini-Mental Status Examination

Table S2c Maximum life-time income and the risk of incident frailty at 14-year follow-up, women (n=256)

	Model 1	Model 2	Model 3	Model 4	Model 5	Model 6	Model 7	Model 8
Variables	OR (95% CI)	OR (95% CI)	OR (95% CI)	OR (95% CI)	OR (95% CI)	OR (95% CI)	OR (95% CI)	OR (95% CI)
Socio-demographics								
Maximum life-time income (ref= Quartile 3 and 4 (HKD$8,000-13,999 and ≥HKD$14,000^))								
Quartile 2 HKD$2,500-7900	1.72 (0.86-3.45)	1.73 (0.86-3.49)	1.73 (0.84-3.58)	1.64 (0.79-3.41)	1.90 (0.90-3.99)	1.89 (0.88-4.06)	1.91 (0.89-4.13)	1.98 (0.91-4.29)
Quartile 1 HKD$0-2,499	1.49 (0.78-2.87)	1.49 (0.78-2.87)	1.50 (0.75-3.00)	1.46 (0.73-2.92)	1.56 (0.77-3.15)	1.57 (0.76-3.25)	1.57 (0.76-3.25)	1.58 (0.77-3.28)
Age	1.25 (1.16-1.35)	1.25 (1.16-1.36)	1.25 (1.16-1.36)	1.26 (1.16-1.36)	1.25 (1.15-1.35)	1.24 (1.14-1.35)	1.24 (1.14-1.35)	1.25 (1.14-1.36)
Non-married (single, divorced, separated) (ref=Married)		0.98 (0.56-1.71)	0.99 (0.56-1.75)	1.01 (0.57-1.79)	0.96 (0.54-1.71)	0.98 (0.55-1.75)	0.98 (0.55-1.75)	0.97 (0.54-1.74)
Educational level (ref=At least completed primary)								
Some primary			1.02 (0.55-1.91)	0.97 (0.52-1.82)	1.01 (0.53-1.91)	0.95 (0.50-1.83)	0.95 (0.50-1.83)	1.00 (0.52-1.94)
No education			0.96 (0.48-1.92)	0.95 (0.47-1.91)	1.15 (0.56-2.37)	1.03 (0.49-2.15)	1.02 (0.49-2.15)	1.26 (0.55-2.89)
Socioeconomic status								
Subjective social status (ref=High)								
Low to middle*				1.45 (0.84-2.51)	1.56 (0.89-2.72)	1.60 (0.91-2.82)	1.61 (0.91-2.83)	1.61 (0.91-2.84)
Medical history								
Hypertension (ref=No hypertension)					2.08 (1.19-3.65)	2.00 (1.12-3.58)	1.99 (1.11-3.57)	2.03 (1.13-3.64)
Diabetes (ref=No diabetes)					1.60 (0.73-3.48)	1.50 (0.68-3.30)	1.49 (0.67-3.27)	1.45 (0.66-3.21)
Stroke (ref=No stroke)					5.70 (0.81-40.04)	6.67 (0.93-47.74)	6.44 (0.89-46.55)	6.72 (0.93-48.44)
Lifestyle								
Current smoker (ref=Non-current						0.62 (0.05-8.10)	0.62 (0.05-8.07)	0.52 (0.04-6.64)

smoker)								
Current drinker (ref=Non –current drinker)						1.22 (0.70-2.15)	1.21 (0.69-2.13)	1.24 (0.70-2.19)
Physical activity, PASE score						1.00 (0.99-1.01)	1.00 (0.99-1.01)	1.00 (0.99-1.01)
Diet quality, DQI						0.99 (0.97-1.02)	0.99 (0.97-1.02)	0.99 (0.96-1.02)
BMI, kg/m^2						1.08 (0.98-1.19)	1.08 (0.98-1.18)	1.08 (0.98-1.19)
Mental health								
SF-12 MCS score							1.01 (0.97-1.05)	1.01 (0.97-1.06)
Cognitive function								
MMSE score								1.06 (0.96-1.16)

^ Maximum life-time income of quartile 3 and 4 (HKD$8,000-13,999 and ≥HKD$14,000) were combined due to small sample sizes
* Subjective social status of low and middle groups were combined due to small sample sizes
Model 1 was adjusted for age at baseline
Model 2 was adjusted for variable in model 1 plus marital status at baseline
Model 3 was adjusted for variables in model 2 plus educational level at baseline
Model 4 was adjusted for variables in model 3 plus subjective social status (community status ladder) at baseline
Model 5 was adjusted for variables in model 4 plus medical history (hypertension, diabetes, and stroke) at baseline
Model 6 was adjusted for variables in model 5 plus lifestyle (smoking status, alcohol consumption, physical activity (PASE score), diet quality (DQI), BMI) at baseline
Model 7 was adjusted for variables in model 6 plus mental health (SF-12 MCS) at baseline
Model 8 was adjusted for variables in model 7 plus cognitive function (MMSE) at baseline
Ref, Reference; PASE, Physical Activity Scale of the Elderly; DQI, Diet Quality Index; BMI, Body Mass Index; SF-12 MCS, Short Form-12 Mental Component Summary; MMSE, Mini-Mental Status Examination

Table S3 Baseline characteristics of the study population retained in the 14-year follow-up versus those loss to follow-up (n=4000)

	Loss to follow-up (n=2939)	Retained in the study (n=1061)	
	Mean ± SD / n (%)	Mean ± SD / n (%)	P
Demographics			
Age, years	73.4±5.3	70.0±3.8	<0.001
Sex			
Men	1491 (50.7)	509 (48.0)	0.124
Women	1448 (49.3)	552 (52.0)	
Marital status			
Married	2005 (68.2)	822 (77.5)	<0.001
Non-married (single, divorced, separated)	933 (31.8)	239 (22.5)	
Objective socioeconomic status			
Educational level			
At least completed primary	1257 (42.8)	563 (53.1)	<0.001
Some primary	1004 (34.2)	320 (30.2)	
No education	678 (23.1)	178 (16.8)	
Maximum life-time income			
Quartile 4 ≥HKD$14,000	390 (18.9)	220 (26.0)	<0.001
Quartile 3 HKD$8,000-13,999	380 (18.4)	203 (24.0)	
Quartile 2 HKD$2,500-7900	641 (31.0)	208 (24.6)	
Quartile 1 HKD$0-2,499	657 (31.8)	214 (25.3)	
Medical history			
Hypertension			
No	1643 (55.9)	650 (61.3)	0.002
Yes	1296 (44.1)	411 (38.7)	
Diabetes			
No	2484 (84.5)	937 (88.3)	0.003
Yes	455 (15.5)	124 (11.7)	
Stroke			
No	2803 (95.4)	1022 (96.3)	0.194
Yes	136 (4.6)	39 (3.7)	
Lifestyle			
Current smoker			
No	2711 (92.2)	1014 (95.6)	<0.001

Yes	228 (7.8)	47 (4.4)	
Current drinker			
No	1415 (48.1)	477 (45.0)	0.075
Yes	1524 (51.9)	584 (55.0)	
Physical activity, PASE score	87.9±41.4	100.8±45.9	<0.001
Diet quality, DQI	63.9±9.7	65.6±9.1	<0.001
BMI, kg/m^2	23.6±3.4	23.8±3.0	0.117
Mental health			
SF-12 MCS score	55.2±7.50	56.11±6.65	<0.001
Cognitive function			
MMSE score	25.2±3.82	26.7±3.03	<0.001
Subjective social status			
High	1486 (53.1)	609 (58.8)	<0.001
Middle	1001 (35.8)	357 (34.5)	
Low	312 (11.1)	70 (6.8)	
Frailty status			
Robust	1426 (48.5)	712 (67.1)	<0.001
Pre-frail	1325 (45.1)	334 (31.5)	
Frail	188 (6.4)	15 (1.4)	

Missing data: Marital status (Loss to follow-up, n=1); Maximum life-time income (Loss to follow-up, n=871; Retained in the study, n=216); Subjective social status (Loss to follow-up, n=140; Retained in the study, n=25)

PASE, Physical Activity Scale of the Elderly; DQI, Diet Quality Index; BMI, Body Mass Index; SF-12 MCS, Short Form-12 Mental Component Summary; MMSE, Mini-Mental Status Examination
